# Serum omentin and chemerin levels in patients with coronavirus disease 2019

**DOI:** 10.3389/fmed.2026.1785101

**Published:** 2026-03-18

**Authors:** Tomasz Maksymilian Wikar, Michał Zdzisław Kukla, Dominika Stygar, Elżbieta Chełmecka, Michał Wysocki, Barbara Maziarz, Mateusz Rubinkiewicz

**Affiliations:** 1 2nd Department of General Surgery, Jagiellonian University Medical College, Kraków, Poland; 2 Faculty of Medicine, Jagiellonian University Medical College, Kraków, Poland; 3 Department of Endoscopy, University Hospital in Kraków, Katowice, Poland; 4 Department of Physiology, Faculty of Medical Sciences in Zabrze, Medical University of Silesia, Zabrze, Poland; 5 Department of Medical Statistic, Faculty of Pharmaceutical Sciences in Sosnowiec, Medical University of Silesia, Sosnowiec, Poland; 6 Department of General Surgery and Surgical Oncology, Ludwik Rydygier Memorial Hospital, Katowice, Poland; 7 Department of Diagnostics, University Hospital, Kraków, Poland

**Keywords:** adipokines, chemerin, COVID-19, inflammation, obesity, omentin, SARS-CoV-2

## Abstract

**Background:**

Chemerin and omentin are adipokines secreted mainly by visceral adipose tissue, with pro- and anti-inflammatory properties, respectively. Their role in coronavirus disease 2019 (COVID-19) remains incompletely understood and available data are inconsistent.

**Methods:**

This single-center case-control study included 40 hospitalized patients with COVID-19 and 24 non-COVID controls. Serum samples were collected in COVID-19 patients on admission (Day 0) and on Day 7 of hospitalization, and once in controls. Concentrations of omentin and chemerin and routine laboratory parameters were measured using enzyme immunoassays.

**Results:**

Compared with controls, patients with COVID-19 had higher inflammatory markers, including C-reactive protein, ferritin, interleukin-6 and D-dimer. Baseline serum omentin concentrations did not differ between COVID-19 patients and controls (363.6 [245.2–513.0] vs. 368.9 [254.1–468.8] ng/mL; *p* = 0.994), and remained stable between Day 0 and Day 7 (*p* = 0.605). In contrast, baseline chemerin levels were significantly higher in COVID-19 patients than in controls (234.3 [164.9–269.9] vs. 144.7 [98.0–213.2] ng/mL; *p* = 0.001) and remained elevated at Day 7 (243.7 [171.0–376.7] ng/mL, *p* = 0.001 vs. controls), with a non-significant trend toward an increase over time (*Δ* chemerin +42.6 ng/mL; *p* = 0.071).

**Conclusion:**

In this cohort of hospitalized but predominantly non-critically ill patients, COVID-19 was associated with sustained elevation of circulating chemerin but not with alterations in omentin levels. Our findings are consistent with a potential role for chemerin, but not omentin, in the systemic inflammatory response to SARS-CoV-2 infection and complement previous reports describing divergent adipokine profiles in COVID-19.

## Introduction

Obesity and visceral adiposity have emerged as major risk factors for severe coronavirus disease 2019 (COVID-19), with excess adipose tissue contributing to worse respiratory outcomes, need for intensive care and mortality (1–4). Adipose tissue is now recognized as an active endocrine organ that produces a broad spectrum of bioactive mediators termed adipokines. These molecules modulate glucose and lipid metabolism, vascular function, and innate and adaptive immunity, and have been implicated in cardiometabolic disease and acute and chronic inflammation (2, 5–8). In the context of SARS-CoV-2 infection, dysregulated adipokine secretion has been proposed as one of the mechanisms linking obesity with impaired antiviral immunity, hyperinflammation and endothelial dysfunction (1–4).

Among the numerous adipokines, chemerin and omentin are of particular interest because of their opposite biological profiles. Chemerin is a chemoattractant ligand for ChemR23 (CMKLR1) that promotes recruitment of dendritic cells and macrophages, modulates endothelial activation, and participates in both initiation and resolution of inflammation (6, 7, 9). Elevated chemerin levels have been reported in several cardiometabolic and inflammatory conditions and correlate with markers of systemic inflammation (9). In contrast, omentin is predominantly expressed in visceral adipose tissue and exerts insulin-sensitizing, vasculoprotective and anti-inflammatory effects, partly via enhancement of nitric oxide bioavailability and suppression of nuclear factor-κB and interleukin-6 signaling (5, 6, 10, 11). Circulating omentin concentrations are typically reduced in obesity, type 2 diabetes and metabolic syndrome (5, 6, 11).

Several studies have examined chemerin and omentin in COVID-19, but their results are conflicting. Kukla et al. (12) reported significantly lower serum chemerin and omentin concentrations in COVID-19 patients compared with healthy controls and found associations with liver injury and metabolic abnormalities. In a case-control study from Iraq, chemerin levels increased with COVID-19 severity, whereas omentin showed negative gradient (13). A recent review summarizing the network of adipocytokines in COVID-19 also emphasized that chemerin and omentin may be decreased in SARS-CoV-2 infection, although data are scarce and heterogeneous (14). On the other hand, Lavis et al. (15) demonstrated that higher chemerin serum levels independently predicted mortality in hospitalized COVID-19 patients, a finding that was broadly corroborated by another cohort in which chemerin was a risk factor for adverse outcomes (16). Conversely, Pavel et al. (17) observed that serum chemerin levels in COVID-19 were more strongly influenced by underlying comorbidities than by acute infection itself. Additional work has suggested that chemerin, adiponectin and leptin may predict clinical course and post-COVID pulmonary sequelae (18).

Evidence regarding omentin in COVID-19 is even more limited. A systematic review of organokines in COVID-19 identified only one small pilot study including omentin, which suggested decreased levels in infected patients compared with controls (2). Moreover, experimental and clinical studies outside the COVID-19 setting consistently support an anti-inflammatory and cardioprotective role of omentin (5, 6, 10, 11), raising the hypothesis that reduced omentin might contribute to endothelial dysfunction and thromboinflammation in SARS-CoV-2 infection.

**Figure 1:**
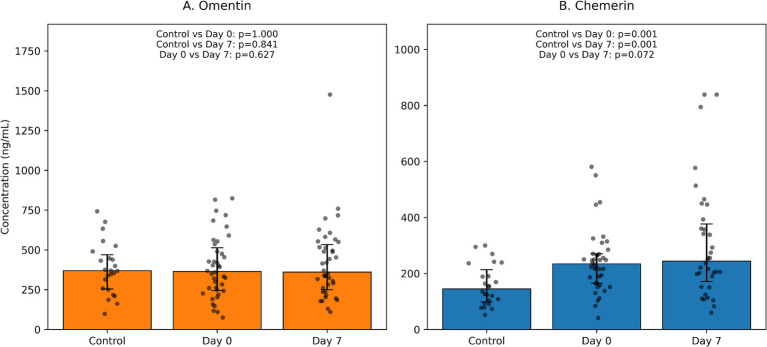
Serum omentin **(A)** and chemerin **(B)** concentrations in COVID-19 patients and controls. Bars represent medians with interquartile ranges (IQR); dots indicate individual data points. Between-group comparisons (COVID-19 vs. controls) were performed using the Mann–Whitney *U* test; within-patient day 0 vs. day 7 comparisons were performed using the Wilcoxon signed-rank test. *p*-values for control vs. day 0, control vs. day 7, and day 0 vs. day 7 comparisons are shown in each panel.

In the current work, we focus on two adipokines with predominantly anti-inflammatory (omentin) and mixed pro-/pro-resolving (chemerin) properties. We aimed to compare serum omentin and chemerin levels between hospitalized patients with COVID-19 and non-COVID controls, to explore short-term changes in these adipokines during the first week of hospitalization, and to relate them to standard inflammatory and biochemical markers (Figure 1).

## Materials and methods

### Study design and population

This study is an analysis of an observational cohort of adults hospitalized with COVID-19. Forty consecutive patients admitted to a tertiary care hospital with reverse-transcriptase polymerase chain reaction (RT-PCR)-confirmed SARS-CoV-2 infection and symptomatic disease were included. The control group consisted of 24 patients scheduled for elective cholecystectomy due to uncomplicated cholelithiasis, without chronic liver or systemic inflammatory diseases. Because gallstone disease can be associated with metabolic risk factors, we report detailed baseline anthropometrics and metabolic laboratory parameters for both groups and interpret adipokine comparisons in this context. All participants were aged ≥18 years. Key exclusion criteria in both groups were chronic viral hepatitis, human immunodeficiency virus co-infection, active malignancy, chronic kidney disease, significant psychiatric illness and known autoimmune liver disease. All patients were managed according to the established therapy protocol, including dexamethasone 6 mg daily, therapeutic-dose low-molecular-weight heparin. Remdesivir was administered when onset of symptoms was less than 5 days before hospitalization. Antibiotics (ceftriaxone 1.0 g twice daily plus ciprofloxacin 0.4 g twice daily) were used in patients with radiologic pneumonia and leukocytosis. Overall, disease severity in the cohort was mild to moderate (Table 1).

**Table 1:** Baseline biochemical characteristics of COVID-19 patients and controls.

Parameter	COVID-19 (Day 0), median (Q1–Q3)	Control group, median (Q1–Q3)	*p*-value
ALT (U/L)	22.5 (16–53)	18 (15.5–27)	0.199
AST (U/L)	33 (22–51)	22 (19.5–28)	0.015
GGT (U/L)	32 (18.5–82)	18 (14.5–26.5)	0.014
Creatinine (μmol/L)	67.85 (57.3–89.3)	71.85 (64.15–83.6)	0.462
Glucose (mmol/L)	5.78 (4.9–6.96)	5.12 (4.78–5.38)	0.017
Ferritin (μg/L)	446 (225.5–657)	85.5 (55–143.5)	<0.001
IL-6 (pg/mL)	30 (16.1–92.14)	1.5 (1.5–2.29)	<0.001
CRP (mg/L)	52.75 (28.85–123.5)	1.28 (0.5–3.7)	<0.001
Procalcitonin (ng/mL)	0.13 (0.02–0.55)	0.02 (0.01–0.04)	0.033
ALP (U/L)	64.5 (46.5–90.5)	67 (59–75)	0.961
LDH (U/L)	231.5 (185.5–328)	188 (174–210)	0.007
Total bilirubin (μmol/L)	8.2 (6.06–11.95)	7.69 (6.79–11.5)	0.771
Ammonia (μmol/L)	34.55 (25.6–45.85)	32.8 (22.1–40.3)	0.501
INR	1.02 (0.96–1.23)	0.96 (0.91–1)	0.003
D-dimer (mg/L)	1.49 (0.77–4.22)	0.3 (0.22–0.35)	<0.001
Sodium (mmol/L)	138 (136–140)	140 (138.5–141)	0.009
Potassium (mmol/L)	4.27 (3.94–4.64)	4.33 (4.21–4.58)	0.279
Urea (mmol/L)	5.9 (4.32–7.94)	4.64 (3.79–5.45)	0.020
Lactate (mmol/L)	1.25 (0.85–1.7)	1.2 (0.8–1.3)	0.419
Creatine kinase (U/L)	67 (44.5–137.5)	76 (61–95.5)	0.857
Troponin (ng/L)	6.06 (2.5–26.3)	3 (3–3.39)	0.020
Myoglobin (ng/mL)	56.3 (34.85–105.2)	37 (21.7–44.6)	0.042
Total cholesterol (mmol/L)	3.2 (2.65–4.25)	4.6 (4.05–5.4)	<0.001
HDL cholesterol (mmol/L)	0.96 (0.74–1.15)	1.43 (1.17–1.62)	<0.001
LDL cholesterol (mmol/L)	1.45 (1.1–2.4)	2.5 (2.05–3)	0.001
Triglycerides (mmol/L)	1.17 (0.89–1.6)	1.07 (0.85–1.77)	0.917
Total protein (g/L)	61 (54.5–65.7)	72.1 (66.9–73.6)	<0.001
Albumin (g/L)	34.4 (29.7–39.75)	46.45 (43.8–48.45)	<0.001

The study was conducted in accordance with the Declaration of Helsinki and approved by the Ethics Committee of the Jagiellonian University in Cracow (resolution number 1072.6120.157.2020).

### Data collection and routine laboratory measurements

Demographic data, comorbidities and basic clinical parameters were obtained from electronic medical records. Standard laboratory tests performed at admission included complete blood count, liver enzymes, renal function tests, coagulation parameters, C-reactive protein (CRP), procalcitonin, ferritin, lactate dehydrogenase (LDH), D-dimer, cardiac biomarkers and basic lipid profile. Selected biochemical parameters (alanine aminotransferase, aspartate aminotransferase, *γ*-glutamyltransferase, creatinine, glucose, ferritin and interleukin-6) were reassessed on day 7 of hospitalization in the COVID-19 group. The control group had routine laboratory tests performed once at baseline. All assays were carried out in the hospital’s central laboratory using validated automated methods.

### Measurement of omentin and chemerin

Residual serum samples obtained at Day 0 and Day 7 from COVID-19 patients and baseline samples from controls were stored at −80 °C until analysis. Serum concentrations of chemerin and omentin-1 were measured in duplicate using sandwich enzyme-linked immunosorbent assay (ELISA) kits (BioVendor, Brno, Czech Republic) (Chemerin Human ELISA, Cat. No. RD191136200R; Omentin-1 Human ELISA, Cat. No. RD191100200R) according to the manufacturer’s instructions. For chemerin, the calibration range was 0.25–8 ng/mL with a limit of detection of 0.1 ng/mL (sample volume 5 μL/well; intra-assay CV 6.0% and inter-assay CV 7.6%). For omentin-1, the calibration range was 2–64 ng/mL with a limit of detection of 0.5 ng/mL (sample volume 6 μL/well; intra-assay CV 3.7% and inter-assay CV 4.6%). All samples from a given participant were analyzed on the same plate to minimize inter-assay variability.

### Statistical analysis

Continuous variables are presented as medians with interquartile ranges (Q1–Q3), and categorical variables as counts and percentages. The Shapiro–Wilk test was used to assess normality. Between-group differences in continuous variables (COVID-19 vs. controls; independent samples) were evaluated using the Wilcoxon rank-sum test (Mann–Whitney *U*). Within-patient changes between Day 0 and Day 7 in the COVID-19 group (paired samples) were analyzed with the Wilcoxon signed-rank test. Thus, both comparisons used rank-based nonparametric tests, selected according to whether samples were independent or paired. Analyses were unadjusted; given the limited sample size, we did not perform multivariable models adjusting for metabolic comorbidities, age, or medication exposure to avoid overfitting. For selected variables we additionally calculated the change (Delta = Day 7 − Day 0) and described it as median (Q1–Q3). A two-sided *p*-value < 0.05 was considered statistically significant. All analyses were performed using STATISTICA 10.0 (StatSoft, Cracow, Poland).

## Results

### Baseline characteristics

The study included 64 participants: 40 patients with COVID-19 (62.5%) and 24 controls (37.5%). Patients with COVID-19 were older than controls (median age 63 [37–76] vs. 48 [34–59] years; *p* = 0.016), whereas sex distribution, waist circumference and body mass index did not differ significantly between groups (all *p* > 0.2). COVID-19 patients had lower hemoglobin, hematocrit and red blood cell counts and showed leukocyte alterations, with higher neutrophil and lower lymphocyte counts compared with controls (all *p* < 0.001). Platelet counts were modestly reduced in COVID-19 (median 202.5 vs. 259.5 × 10^9^/L; *p* = 0.005).

Markers of systemic inflammation and coagulopathy were markedly elevated in COVID-19 patients. Median CRP (52.75 vs. 1.28 mg/L), ferritin (446 vs. 85.5 μg/L), D-dimer (1.49 vs. 0.30 mg/L) and interleukin-6 (30 vs. 1.5 pg./mL) were all significantly higher in the COVID-19 group than in controls (all *p* < 0.001). COVID-19 patients also had higher NT-proBNP and cardiac troponin concentrations and lower total cholesterol, LDL cholesterol and HDL cholesterol values than controls. Liver enzymes were largely within the reference range but alanine and aspartate aminotransferase activities tended to be higher in the COVID-19 group.

### Serum omentin levels in COVID-19 patients and controls

Baseline serum omentin concentrations (Day 0) were very similar in COVID-19 patients and controls. Median omentin levels were 363.6 (245.2–513.0) ng/mL in the COVID-19 group and 368.9 (254.1–468.8) ng/mL in the control group (*p* = 0.994). On Day 7 of hospitalization, omentin levels in COVID-19 patients remained comparable to baseline, with a median of 360.8 (249.4–533.8) ng/mL. The within-patient change in omentin between Day 0 and Day 7 was negligible (*Δ* − 1.1 [−90.8; 118.6] ng/mL; *p* = 0.605). Taken together, these data indicate that acute, non-critical COVID-19 did not significantly alter circulating omentin concentrations in this cohort.

### Serum chemerin levels in COVID-19 patients and controls

In contrast to omentin, chemerin levels differed substantially between groups. At baseline, median chemerin concentrations were 234.3 (164.9–269.9) ng/mL in COVID-19 patients and 144.7 (98.0–213.2) ng/mL in controls (*p* = 0.001). On day 7, chemerin levels in the COVID-19 group remained elevated, with a median of 243.7 (171.0–376.7) ng/mL, and were still significantly higher than in controls (*p* = 0.001 vs. control values). Within the COVID-19 group, chemerin showed a trend toward an increase during hospitalization, with a median *Δ* of +42.7 (−99.7; 180.5) ng/mL and a borderline *p*-value of 0.071. These findings suggest a sustained activation of the chemerin axis in the early course of COVID-19, with possible further up-regulation over time.

## Discussion

In this analysis of a cohort of hospitalized adults with COVID-19 and non-COVID controls, we observed a clear dissociation between two adipokines derived predominantly from visceral adipose tissue. Circulating chemerin levels were significantly higher in COVID-19 patients than in controls at baseline and remained elevated after 1 week of hospitalization, with a trend toward further increase. In contrast, serum omentin concentrations were similar in patients and controls and remained stable over time. These results are consistent with a selective involvement of chemerin, but not omentin, in the systemic inflammatory response to SARS-CoV-2 infection in predominantly non-critically ill patients.

Our findings add to a growing but heterogeneous body of literature on chemerin in COVID-19. Kukla et al. (12) reported lower chemerin concentrations in COVID-19 patients compared with healthy volunteers, whereas several subsequent studies, including the cohorts analysed by Lavis et al. (15) and Gökdemir et al. (16) found higher chemerin levels to be associated with disease severity and mortality. Pavel et al. (17) observed that chemerin levels in hospitalized patients with COVID-19 were influenced more strongly by underlying comorbidities than by the acute infection itself. The elevated chemerin concentrations in our cohort, together with markedly increased conventional inflammatory markers, support the concept that chemerin behaves as an acute-phase reactant and may reflect systemic inflammation and endothelial activation in COVID-19 (9, 15–18). The lack of ICU-level illness in most of our patients likely explains why chemerin did not show a clear gradient according to disease severity.

Data on omentin in COVID-19 remain limited and inconsistent. In the Scientific Reports cohort analysed by Kukla et al. (12) both chemerin and omentin were reduced in COVID-19 patients relative to controls, and a recent review of organokines in COVID-19 highlighted that omentin levels were decreased in the only available pilot study (2, 14). In contrast, we did not observe any difference in omentin between COVID-19 patients and controls, nor any significant short-term change during hospitalization. Several factors may account for these discrepancies, including differences in patient selection (ours were relatively lean, with few individuals with class II or III obesity), timing of sampling, assay characteristics, concomitant medications such as corticosteroids, and underlying comorbidities. It is also possible that omentin plays a more prominent role in severe or critical COVID-19, or in patients with substantial metabolic derangements, than in the predominantly mild-to-moderate cases studied here.

Elevated chemerin in our cohort is biologically plausible. Chemerin is up-regulated by pro-inflammatory cytokines, including interleukin-6 and tumor necrosis factor-*α*, and can act on immune cells and endothelial cells to amplify or modulate inflammatory responses (6, 7, 9). COVID-19 is characterized by a cytokine-rich milieu and widespread endothelial activation, even in patients with non-critical disease (1–4). Chemerin may therefore participate in leukocyte recruitment to inflamed tissues and in thromboinflammatory processes, potentially contributing to microvascular dysfunction and organ damage (9, 15–18). In contrast, omentin has primarily anti-inflammatory and vasculoprotective actions, with lower circulating levels typically observed in obesity, insulin resistance and cardiometabolic disorders (5, 6, 10, 11). The preserved omentin levels in our patients may reflect the relatively modest burden of obesity and metabolic disease in the cohort, as well as the short observation window.

Our study also needs to be interpreted in the context of the previously reported by us visfatin and leptin data (19). We have shown that visfatin levels were markedly reduced at admission and returned to control values by day 7, whereas leptin levels did not differ significantly between patients and controls (19). Together with the present findings, this suggests that SARS-CoV-2 infection induces a complex and adipokine-specific pattern of responses, with some mediators (visfatin) showing transient depletion, others (chemerin) exhibiting sustained elevation, and others (leptin, omentin) remaining largely unchanged. This heterogeneity likely reflects differences in tissue sources, receptor distribution, regulation by cytokines and glucocorticoids, and downstream signaling pathways.

Strengths of our work include the use of a well-defined patient and control group, standardized sample collection at two clearly defined time points, and the concurrent assessment of a broad panel of routine laboratory parameters. However, several limitations must be acknowledged. First, the relatively small sample size limits statistical power, especially for detecting modest within-patient changes and for subgroup analyses according to body mass index or disease severity; importantly, no *a priori* power calculation was performed, so non-significant results should be interpreted cautiously as potentially reflecting limited power (type II error). This increases the risk of a type II error (*β*), i.e., failing to detect a true difference or within-patient change (a false-negative result). Moreover, because the clinical course in our cohort was predominantly mild-to-moderate, there were no ICU admissions or deaths, precluding assessment of adipokines in relation to these outcomes. Additionally, adipokines were assessed at only two time points (Day 0 and Day 7), which may not fully capture their temporal dynamics during later stages of illness or recovery. Second, the control group consisted of patients with uncomplicated cholelithiasis undergoing elective cholecystectomy. Gallstone disease is frequently linked to obesity, insulin resistance and other components of the metabolic syndrome, which could influence circulating adipokine concentrations. Therefore, residual metabolic confounding cannot be fully excluded and may have attenuated or biased between-group differences, despite comparable BMI and the absence of chronic liver or systemic inflammatory diseases in controls. In addition, because COVID-19 patients and controls differed in age and may have differed in the prevalence of metabolic comorbidities (e.g., diabetes) and in medication use, residual confounding cannot be excluded; we therefore interpret between-group comparisons as exploratory and hypothesis-generating. Moreover, information on in-hospital corticosteroid or other anti-inflammatory/immunomodulatory treatments was not systematically analyzed, and such therapies could have influenced adipokine concentrations over time. Third, we did not have detailed data on visceral adipose tissue volume or longitudinal clinical outcomes such as thromboembolic events, which precludes exploration of chemerin and omentin as prognostic biomarkers. Finally, the study was conducted during the early phase of the pandemic, before widespread vaccination and emergence of later SARS-CoV-2 variants, which may limit generalizability to current clinical settings.

Despite these limitations, our results provide novel information on the behavior of two biologically relevant adipokines in COVID-19. Future studies in larger, prospectively followed populations should incorporate more frequent serial measurements of chemerin and omentin across acute and convalescent phases, detailed imaging of body fat distribution, and robust clinical endpoints to determine whether these adipokines add prognostic value beyond standard inflammatory and cardiometabolic markers.

## Conclusion

In hospitalized adults with predominantly mild-to-moderate COVID-19, serum chemerin levels were significantly elevated compared with non-COVID controls and remained high during the first week of hospitalization, whereas circulating omentin concentrations were similar in patients and controls and showed no meaningful short-term change. These findings are consistent with a role for chemerin, but not omentin, as a component of the systemic inflammatory response to SARS-CoV-2 infection. Further research is needed to clarify whether chemerin may serve as a biomarker of disease severity or therapeutic response in COVID-19 and to better understand the determinants and consequences of omentin dynamics in this setting.

## Data Availability

The raw data supporting the conclusions of this article will be made available by the authors, without undue reservation.

## References

[1] Rychter AM Zawada A Ratajczak AE Dobrowolska A Krela-Kaźmierczak I. Should patients with obesity be more afraid of COVID-19? Obes Rev. (2020) 21:e13083. doi: 10.1111/obr.13083, 32583537 PMC7362042

[2] Barbalho SM Haber JF Tofano RJ Serrat J Torres-Valle M López-García M . Molecular characterization of the interplay between *Fasciola hepatica* juveniles and Laminin as a mechanism to adhere to and break through the host Intestinal Wall. Int J Mol Sci. (2023) 24:8165. doi: 10.3390/ijms24098165, 37175870 PMC10179147

[3] Grewal T Buechler C. Adipokines as diagnostic and prognostic markers for the severity of COVID-19. Biomedicine. (2023) 11:1302. doi: 10.3390/biomedicines11051302, 37238973 PMC10215701

[4] Belchior-Bezerra M Lima RS Medeiros NI Gomes JAS. COVID-19, obesity, and immune response 2 years after the pandemic: A timeline of scientific advances. Obes Rev. (2022) 23:e13483. doi: 10.1111/obr.13496 35837843 PMC9349458

[5] Halabis M Dziedzic M Warchulińska J Kaznowska-Bystryk I Solski J. Omentin - a new adipokine with many roles to play. Current Issues in Pharmacy and Medical Sciences. (2015) 28:176–180. doi: 10.1515/cipms-2015-0067

[6] Radzik-Zając J Wytrychowski K Wiśniewski A Barg W. The role of the novel adipokines vaspin and omentin in chronic inflammatory diseases. Pediatr Endocrinol Diabetes Metab. (2023) 29:48–52. doi: 10.5114/pedm.2022.121371 36734393 PMC10226453

[7] Jung HN Jung CH. The Role of Anti-Inflammatory Adipokines in Cardiometabolic Disorders: Moving beyond Adiponectin. Int J Mol Sci. (2021) 22:13529. doi: 10.3390/ijms222413529 34948320 PMC8707770

[8] Shaw A Tóth BB Király R Arianti R Csomós I Póliska S . Irisin stimulates the release of CXCL1 from differentiating human subcutaneous and deep-neck derived adipocytes via upregulation of NFκB pathway. Front Cell Dev Biol. (2021) 9:737872. doi: 10.3389/fcell.2021.737872, 34708041 PMC8542801

[9] Acewicz M Kasacka I. Chemerin activity in selected pathological states of human body - A systematic review. Adv Med Sci. (2021) 66, 270–278. doi: 10.1016/j.advms.2021.05.002 34082283

[10] Tan YL Zheng XL Tang CK. The protective functions of omentin in cardiovascular diseases. Clin Chim Acta. (2015) 448:98–106. doi: 10.1016/j.cca.2015.05.019 26079253

[11] Biegański HM Dąbrowski KM Różańska-Walędziak A . Omentin—General Overview of Its Role in Obesity, Metabolic Syndrome and Other Diseases; Problem of Current Research State. Biomedicines. (2025) 13:632. doi: 10.3390/biomedicines13030632 40149608 PMC11940803

[12] Kukla M Adamek B Waluga M Menżyk T Dembiński M Winiarski M . Anti-inflammatory adipokines: chemerin, vaspin, omentin concentrations and SARS-CoV-2 outcomes. Sci Rep. (2021) 11:2283. doi: 10.1038/s41598-021-00928-w, 34728695 PMC8563971

[13] Lama H Ali R Adday Ali H Naser R Majeed A Rufaie AM . Assessment of the serum levels of chemerin and Omentin in among Iraqi patients as early Predictors markers of the Severity COVID-19. (2022).

[14] Nigro E D’Agnano V Quarcio G Mariniello DF Bianco A Daniele A . Exploring the Network between Adipocytokines and Inflammatory Response in SARS-CoV-2 Infection: A Scoping Review. Nutrients. (2023) 15:3806. doi: 10.3390/nu15173806 37686837 PMC10490077

[15] Lavis P Ducastel M de Van Borne P . Chemerin serum levels are increased in COVID-19 patients and are an independent risk factor of mortality. Front Immunol. (2022) 13:942125. doi: 10.3389/fimmu.2022.941663, 36032171 PMC9412239

[16] Gökdemir GS Bilen Ş Yücel G Gokdemir GŞ Gokdemir MT Araç S . Prognostic significance of the chemerin level in coronavirus disease 2019 patients. Medicine (Baltimore). (2024) 103:e32745. doi: 10.1097/MD.0000000000037743, 38579052 PMC10994447

[17] Pavel V Amend P Schmidtner N Utrata A Birner C Schmid S . Chemerin Levels in COVID-19 Are More Affected by Underlying Diseases than by the Virus Infection Itself. Biomedicines. (2024) 12:2099. doi: 10.3390/biomedicines12092099 39335612 PMC11430512

[18] Esendagli D Esendagli-Yilmaz G Telli F Topcu D Gul E Alperen C . Can adipokines predict clinical prognosis and post-COVID lung sequelae? Cytokine. (2023) 165:156192. doi: 10.1016/j.resinv.2023.06.001, 37433250

[19] Wikar T Rubinkiewicz M Stygar D Chełmecka E Popiela U Michał W . Changes in Circulating Adipokine Levels in COVID-19 Patients. J. Clin. Med. (2024) 13:4784. doi: 10.3390/jcm13164784 39200926 PMC11355170

